# Safe Anæsthesia

**Published:** 1872-01

**Authors:** 


Safe Anæsthesia.—Dr. J. Petrie, of Liverpool, writes to the *British Medical Journal:* "I was among the first in this country who put in use ether as an anæsthetic, after receiving intelligence of its successful employment in Boston, United States. I was then actively engaged as honorary surgeon in the Southern Hospital here; and I, along with my colleagues, continued the use of it, as well as of chloroform, in every operation for many years, without a single death having occurred to us from these anæsthetic agents. Indeed, up to this time, I have never witnessed a death from the inhaling of ether or chloroform, either in my private or hospital practice, although I have used them freely whenever called upon to do so. My object in this communication is merely, at present, to refer to the following passage in your article: 'Is it true that out of all the enormous number of cases in which it (chloroform) has been administered to lying-in women, it has never produced any fatal accident? If so, what is the explanation of such a fact?' In a number of the *Medical Times and Gazette* of 1860 you will find a short letter of mine 'On the Prevention of Accidents from the Inhalation of Chloroform.'

"Seeing the fact on record at the time I wrote, that seventy thousand parturient women had had chloroform during their labor without one death occurring among them, I felt it quite confirmatory of the opinion which I had for some time previously held, and which I still hold, that the position of the patient in the bed, or on the operating table, has everything to do with the immunity from danger in the former case, and with the constantly recurring fatality in the latter. In parturition, as a rule, the woman is placed on her left side, and she inhales the chloroform, with an unmeasured ingress of air, into the lungs, with little or no chance of its being interrupted in any way. The patient undergoing an operation is almost always placed horizontally on his back on the operating table, and inhalations of the chloroform are usually effected by placing a piece of lint, or the corner of a towel sprinkled with chloroform, over the nose and mouth, the apparatus being occasionally lifted to admit air. When the patient is thus rendered insensible, the towel or lint is removed, and respiration may go on, but it not infrequently happens that the tongue, by its weight, falls back, and the epiglottis at its base covers the larynx, and will totally prevent inspiration; so that, under this condition, with the air passages filled with vapor of chloroform, in two or three minutes the patient would inevitably die suffocated, or in an absolute state of apnœa."

